# Effect of Substituting Concentrate Mix With Sweet Potato Vines on Growth Performances and Carcass Components of Yearling Rams and Its Potential in Mitigating Methane Production

**DOI:** 10.1155/vmi/1054348

**Published:** 2025-03-10

**Authors:** Assefa Tadesse, Natascha Titze, Markus Rodehutscord, Aberra Melesse

**Affiliations:** ^1^School of Animal and Range Sciences, Hawassa University, Hawassa, Ethiopia; ^2^Institute of Animal Science, University of Hohenheim, Stuttgart, Germany

**Keywords:** carcass cuts, methane mitigation, potato vine, sheep, weight gain

## Abstract

This study was conducted to investigate the substitution effect of the concentrate mix (CM) with sweet potato vine (SPV) on growth performance, carcass characteristics of rams, and methane (CH_4_) production. Forty yearling rams with an initial body weight of 18.5 ± 0.7 kg were randomly distributed into five treatment diets with eight rams each. A CM of 385 g/head/d was supplied to the control group (SPV0), and diets were formulated to replace the CM of the control diet with SPV at 10% (SPV10), 20% (SPV20), 30% (SPV30), and 40% (SPV40). After 82 days, four rams from each treatment were randomly selected and slaughtered. The CH_4_ production was determined from 24-h *in vitro* gas production (GP), and the digestible organic matter (dOM) and metabolizable energy (ME) were estimated from the GP. Data were analyzed using the one-way ANOVA and orthogonal polynomial contrast functions. Results indicated that the contents of the crude protein (CP) and ash in SPV were 258 and 148 g/kg DM, respectively. The substitution of CM with SPV in the *in vivo* trial did not affect body weight gain, feed intake, and feed conversion ratio (FCR). Rams fed with the SPV0 diet had higher (*p* < 0.05) hot carcass weight than those reared in the SPV10, SPV20, and SPV40 diets. The slaughter weight and dressing percentage showed significant linear and quadratic effects. Moreover, significant linear and cubic trends were noted for hot carcass, thorax, foreleg, and hindleg. The SPV0 diet had the highest GP (*p* < 0.001) compared to the SPV30 and SPV40 diets. The SPV40 diet and the pure SPV had the lowest CH_4_ production (*p* < 0.001). The SPV0 diet showed similar ME with SPV10 and SPV20 diets but was higher (*p* < 0.001) than the SPV30 and SPV40 diets and pure SPV. The dOM did not differ between treatment diets. Similarly, the dOM of SPV did not differ (*p* > 0.05) from that of the SPV30 and SPV40 diets. In conclusion, replacing CM with SPV significantly reduced the *in vitro* CH_4_ production without affecting voluntary feed intake, weight gain, some carcass components, and dOM.

## 1. Introduction

Reducing the greenhouse gas (GHG) emissions from the food production industry is a major challenge in the framework of the projected growth of the world's population, which is associated with the growing demand for food. In this context, the anticipated goal should be to achieve the lowest possible emission intensity in the food production system as measured in the magnitude of GHG emissions per unit of output [1, 2]. One major contributing sector to food production for the growing world population is livestock agriculture which could be based either on pastoralism or as part of mixed cropping–livestock systems. The smallholder mixed crop–livestock systems are particularly important for local and regional food provision and rural development, especially in tropical countries [3]. In the mixed systems, the farm animals are mainly provided with poor-quality forages or crop residues which supply inadequate energy and major nutrients [1]. This has frequently resulted in low performance and live weight losses of farm animals which would affect the food security status of the rural community.

Supplementing the basal feeds that are deficient in major nutrients with either grain concentrate or forage legumes improves the performance of small ruminants. However, in smallholder settings, supplementing poor-quality feeds with grain concentrate is generally inaccessible to many of them. One way of overcoming such gaps would be thus to promote the utilization of unconventional feed resources such as vines of sweet potato (*Ipomoea batatas*) which are produced and left over after harvesting. Using its byproducts notably the leaf and stem parts would solve the problems associated with feed shortage and the cost of feeding and alleviate the pollution problems [4–8]. There are several works reporting on the utilization of sweet potato byproducts such as leaves, vines, and stems for livestock in many African countries [9–11].

According to the reports in [8], the local sweet potato leaves contained relatively high levels of crude protein (CP, 247 g/kg DM), metabolizable energy (ME, 10 MJ/kg DM), and micronutrients, particularly iron and manganese. The same authors have further reported that no soluble condensed tannins were detected in sweet potato leaves, making it a suitable feed resource in livestock nutrition. Animal-based experiments have shown that sweet potato vines (SPVs) are potential alternative feed sources for small ruminants [11, 12]. Melesse, Chalew, and Nurfeta [10] evaluated the effect of sweet potato leaf on the feed intake, growth performance, and digestibility and reported that sheep supplemented with increased levels have gained significantly higher body weights than those in the nonsupplemented group. Moreover, they reported significantly higher apparent digestibility values of organic matter (OM) and CP for all supplemented sheep with improved N retention. Another study conducted [13] revealed that the SPV could be used as a substitute for alfalfa hay up to 100% in the diets of rabbits without affecting growth performance and meat composition.

Tropical pastures are commonly characterized by a high concentration of structural carbohydrates and low CP and energy contents resulting in long ruminal retention times of digesta and in high production of enteric methane (CH_4_) [14, 15]. Under such circumstances, it has been recommended to use the wide variety of plant resources that contain secondary metabolites which might have the potential to mitigate CH_4_ emissions. Among such plant materials, SPV has been reported to be a suitable feed material in mitigating CH_4_ production from livestock agriculture [8]. However, there is limited information available in the literature reporting on the feeding potentials of graded levels of SPV on the growth performances and carcass characteristics, particularly regarding its potential in mitigating the CH_4_ production in the diets of small ruminants. Therefore, this study was undertaken to investigate the effects of substituting the concentrate mix (CM) with different levels of SPV on feed intake, body weight gain, and carcass characteristics of yearling rams and its potential to mitigate the CH_4_ production *in vitro*.

## 2. Materials and Methods

### 2.1. Preparation of Experimental Rations

The sweet potato plant was grown on an experimental farm, and the vines were collected after separating them from the stem. The SPV was then spread on a plastic sheet for drying in an area protected from direct sunlight. After the drying process was completed, the SPV was ground using mortar and pestle, packed in bags of 100 kg, and stored in a cool and dry place until used. The CM was prepared from wheat bran, ground maize, linseed cake, and salt (Table 1). The CM was formulated to contain CP and energy concentrations to meet the minimum recommendation reported in [16] for intensive small ruminant feeding (i.e., 17% CP and 9 MJ ME/kg DM). The SPV was mixed with the CM when preparing the experimental diets.

### 2.2. Experimental Design and Treatment Diets

Forty yearling local rams with an initial average body weight of 18.5 ± 0.7 kg were allowed to acclimatize to the experimental environment for 2 weeks. During the adaptation period, all the rams were sprayed with acaricide and drenched with the recommended dose of anthelmintics. During the acclimatization period, rams were identified with numbered ear tags and fed with a CM and grass hay *ad libitum*. At the end of the acclimatization period, individual animals were weighed and blocked by the similarity of their live weight. Then, individual rams from each block were randomly assigned into five treatment diets with eight rams each. The individual pens were fitted with feeders and watering troughs. The CM/SPV mixture was offered in combination twice a day in equal portions while the grass hay was provided to all treatment groups *ad libitum*. The supply of grass hay was measured, and adjustments were made when the feed refusal was less than 10% of the offered.

The yearling rams were assigned into five dietary groups: SPV0, control diet without SPV; SPV10, a diet with 10% replacement of CM by SPV; SPV20, a diet with 20% replacement of CM by SPV; SPV30, a diet with 30% replacement of CM; and SPV40, a diet with 40% replacement of CM by SPV. Accordingly, the ratios of CM: SPV mixture offered to the SPV0, SPV10, SPV20, SPV30, and SPV40 treatment groups were 385:0, 347:38.5, 308:77, 270:116, and 231:154 g/head day, respectively. The experiment lasted 82 days, excluding the acclimatization period of 15 days.

### 2.3. Data Collection Procedures

#### 2.3.1. Feed Intake and Body Weight

The individual body weight of rams was taken at the start of the experiment and was considered as the initial body weight. To monitor the body weight change, the live weight of individual animals was recorded every fortnight before the morning feed was offered. The amount of feed offered was weighed and recorded every morning while the feed refusal was collected and weighed the next day morning before the feed was offered. Feed intake of CM/SPV and hay was then determined by the difference between the amount of feed offered and that of refused.

At the end of the experiment, all rams were weighed individually in the morning before feeding and this was defined as the final body weight. The total body weight gain was then calculated by subtracting the initial body weight from that of the final. The feed conversion ratio (FCR) was calculated as a ratio of the total feed intake to the total weight gain. Additionally, the individual nutrient intake (DM, CP, NDF, etc.) from hay, CM, and SPV was computed based on their proximate composition and the corresponding total feed intake.

#### 2.3.2. Carcass Components

For the evaluation of carcass components, 20 rams were randomly selected (4 rams from each treatment) and fasted overnight. Prior to the start of slaughtering, the body weight of an individual ram was taken and considered as a slaughter weight. The upper part of the rams' throat was cut by severing the jugular vein and carotid artery with a knife which is a standard and accepted slaughtering procedure of animals in Ethiopia. The skin was properly flayed, and the edible and nonedible carcass components were separately weighed and recorded. The hot carcass weight was determined after the removal of the head, skin, blood, trotters, and all visceral organs. The main carcass cuts included in the hot carcass weight were the thorax, foreleg, hindleg, lumbar, neck, liver, and heart. The dressing percentage was calculated as the proportion of the hot carcass weight to the preslaughter weight. The cross-sectional area of the rib-eye muscle between the 12^th^ and 13^th^ ribs was traced on transparency paper from the right and left side and measured by using a planimeter. The average of the right and left cross-sectional areas was then taken as the rib-eye muscle area.

#### 2.3.3. Ethical Statement

Prior to the start of the experiment, the research proposal of the current work was evaluated and approved by the Graduate Council of the School of Animal and Range Sciences of Hawassa University. A written approval ethical statement was then obtained from the School of Animal and Range Science with reference number ARSc/106/16. The rams were slaughtered in an experimental slaughterhouse using an established slaughtering facility at the School's Animal Farm. The slaughtering process was performed under the supervision of the animal health experts delegated by the School of Veterinary Medicine of Hawassa University by following the local slaughtering procedures with minimum pain exerted on each animal.

### 2.4. Protocols of the In Vitro Gas Production (GP) and Methane Production

#### 2.4.1. GP

The measurements of the *in vitro* GP and CH_4_ production were carried out at the Institute of Animal Science, University of Hohenheim, Germany. The GP was determined according to the procedure of VDLUFA official method (VDLUFA 2007, Method 25.1) and [17]. About 120 mg of feed sample was precisely weighed and transferred into 100-mL calibrated glass syringes fitted with white Vaseline–lubricated glass-made plungers. The buffered mineral solution was prepared and maintained in a water bath at 39°C under continuous flushing with CO_2_. A mixture of rumen fluid was collected from two rumen-fistulated Jersey cows fed a total mixed ration as described in [18, 19]. Three syringes with only buffered rumen fluid referred to as blank (rumen fluid without feed sample), three other syringes containing the hay standard, and another three syringes containing the concentrate standard samples with known GP were also included along with each run. All samples were incubated in a rotary incubator for 24 h, and four independent runs were performed for each feed material and both standards. Immediately after the end of the incubation period, the amount of gas produced from individual syringes was recorded.

#### 2.4.2. Methane Production

After recording the GP, the incubation liquid was decanted carefully, while leaving the gas inside the syringes. The CH_4_ concentration of the total gas in the syringes was then analyzed using an infrared methane analyzer (Pronova Analysentechnik, Berlin, Germany) calibrated with a reference gas (13.0 vol % CH_4_, Westfalen AG, Münster, Germany). The syringes were connected directly to the analyzer, and about 20 mL of gas was injected for about 20 s until the CH_4_ concentration displayed was constant. The CH_4_ produced by each feed sample was corrected by the amount of CH_4_ produced by the blank syringes and by the factors of the reference hay and concentrate feed samples which were included as standards. In addition to the concentrations of CH_4_ (mL/g DM), the ratio of the CH_4_ to the corrected GP was presented as a percentage of the net gas produced. The percentage of CH_4_ (pCH_4_) in the corrected GP was determined according to [8].

#### 2.4.3. Estimation of ME and Digestible Organic Matter (dOM)

For the estimation of ME and dOM, the recorded GP from each feed sample was corrected using the blanks and standards of hay and concentrate. The ME and dOM were then determined according to [17] by using the following formula:

(1) $$\begin{array}{c} \text{ME}\left(\frac{\text{MJ}}{\text{kg}}\text{DM}\right)=1.68+0.1418\times \text{GP}+0.0073\times \text{CP}+0.0217\times \text{XL}-0.0028\text{ XA},\\ \text{dOM}\left(\%\right)=14.88+0.889\times \text{GP}+0.0448\times \text{CP}+0.0651\times \text{XA}, \end{array}$$

where GP (mL/200 mg DM), CP (g/kg DM), XL (g/kg DM), and XA (g/kg DM) are GP, CP, EE, and crude ash, respectively.

### 2.5. Chemical Analysis

Analyses of proximate nutrients and minerals were performed as outlined in [20] at the Institute of Animal Science, University of Hohenheim, Germany. The samples were analyzed for DM (Method 3.1), crude ash (Method 8.1), CP (Method 4.1.1), and petroleum EE (Method 5.1.1). Neutral detergent fiber (aNDFom) was assayed on OM basis after amylase treatment and acid detergent fiber (ADFom) on an OM basis (Methods 6.5.1 and 6.5.2, respectively). Acid detergent lignin (ADL) was analyzed according to Method 6.5.3. The analysis of calcium (Ca), phosphorous (P), magnesium (Mg), potassium (K), sodium (Na), iron (Fe), copper (Cu), and manganese (Mn) were determined according to Methods 10 and 11 of [20] using an inductively coupled plasma spectrometer (ICP-OES). All analyses were performed in duplicate and averaged.

### 2.6. Statistical Analysis

Results of the proximate and mineral compositions are expressed as means for duplicate analysis of feed samples. Data on feed intake, body weight, carcass parts, *in vitro* GP and CH_4_ production, ME, and dOM were subjected to one-way ANOVA by fitting treatment diets as a single factor. The mean comparisons were conducted using Tukey's Studentized range test. In addition, the orthogonal polynomial contrast analysis was performed to determine the linear, quadratic, and cubic trends due to the substitution of CM with SPV on feed intake, weight gain, FCR, and carcass components. All analyses were conducted by using the Statistical Analysis System (SAS, 2016, Ver. 9.4). The effects were considered significant at *p* < 0.05, and variability in the data was expressed as the pooled standard error of the mean (SEM).

## 3. Results

### 3.1. Nutrient Compositions of SPV and Experimental Diets

The analyzed proximate compositions of treatment diets, grass hay, and SPV are presented in Table 2. The CP and ash contents in SPV were higher than those in any of the treatment diets. Similarly, the SPV had higher values of NDFom, ADFom, and ADL than the experimental diets. However, the SPV was low in EE content. Although the grass hay had the lowest values of CP and EE, it contained higher NDFom, ADFom, and ADL as compared with the SPV and experimental diets. The ash content in grass hay was also higher than that in the SPV0, SPV10, and SPV20 diets. Among treatment diets, the ash and CP contents improved with increased substitution levels of CM with SPV. Similarly, the contents of structured carbohydrates consistently elevated with increased substitution levels of SPV. On the other hand, the EE content was reduced with increased substitution levels with SPV.

As shown in Table 3, the SPV had the highest concentrations of Ca and K as compared with grass hay and treatment diets, the latter mineral being exceptionally high. As a result, the concentration of both minerals specifically that of Ca consistently increased across treatment diets with increased replacement of CM by SPV. The concentration of P and Mg was higher in the SPV than in the grass hay, and the former slightly decreased with increased substitution levels. On the other hand, the SPV was deficient in some other minerals, notably Na. The concentration of Cu showed a decreasing trend with increased substitution levels of SPV. The highest Fe concentration was observed in grass hay and showed an inconsistent pattern among treatment diets.

### 3.2. Intake of Nutrients, Body Weight Gain, and Feed Conversion Efficiency

The substitution of CM by SPV up to 40% (SPV40) did not affect the final body weight, weight gain, feed intake, and FCR of the yearling rams (Table 4). Accordingly, the final body weight and the total body weight gain values were similar across treatment diets. The total feed intake and FCR values were also similar across treatment diets. Rams fed with the control diet and the SPV30 diet had similar body weight and weight gain values. Moreover, rams fed on the SPV20 and SPV30 diets showed similar FCR values. None of the parameters studied showed a significant linear, quadratic, or cubic trend with increasing substitution levels of SPV.

As shown in Table 5, the intake of nutrients from hay showed very similar patterns and did not differ among treatment diets. However, the intake of all nutrients from CM significantly (*p* < 0.001) reduced with increased substitution levels of CM by SPV. On the other hand, the intake of all nutrients from SPV consistently increased as the substitution levels of SPV increased. The CP intake from SPV was tripled in the SPV30 and SPV40 diets along with increased substitution levels of CM with SPV. Regarding the total nutrient intake, no difference was observed among treatment diets for DM and NDF intake. As the substitution levels of SPV increased, there was a significant (*p* < 0.001) increase in the total intake of CP and ADF. Conversely, the total EE intake decreased (*p* < 0.001) across treatment diets as the substitution of CM with SPV increased.

### 3.3. Carcass Components

The preslaughter weight has differed (*p* < 0.05) among rams being higher in the SPV0 diet than among those reared in the SPV10, SPV20, and SPV30 diets, while it was similar to those fed on the SPV40 diet (Table 6). Moreover, the preslaughter weight showed linear (*p* = 0.025) and quadratic (*p* = 0.007) effects with increased substitution levels of CM with SPV. Rams fed with the SPV0 diet had also higher (*p* < 0.05) dressed hot carcass weight than those reared in the SPV10, SPV20, and SPV40 diets. Significant linear (*p* = 0003) and cubic (*p* = 0.013) effects were observed in hot carcass weight with increased substitution levels of CM with SPV. The weight of the thorax and foreleg was higher (*p* < 0.05) in rams fed with the SPV0 diet than in those reared in different substitution levels of CM with SPV. The linear, quadratic, and cubic effects were significant for thorax weight with increased substitution levels of CM by SPV. Similarly, significant linear (*p* = 0.002) and cubic (*p* = 0.031) trends were noted for foreleg with increased substitution levels.

Rams fed on the SPV0 diet had higher (*p* < 0.05) hindleg and liver values than those fed with the SPV40 diet. Likewise, significant linear and cubic trends were observed for both carcass cuts (Table 6). The average lumbar weight was highest in rams fed with SPV20 followed by SPV0 though the difference was insignificant. The dressing percentage ranged from 37.6 in rams fed with the SPV0 diet to 39.7% in those reared in the SPV30 diet and was not affected by the substitution of CM with SPV. However, a significant quadratic (*p* = 0.022) trend was observed for dressing percentage with increased substitution levels of CM with SPV. No significant difference was observed for the lumbar, neck, heart, and REA between rams fed with the control diet (SPV0) and the other treatment diets. Similarly, the orthogonal contrast effects were insignificant for these carcass parameters. Although insignificant, rams fed on the SPV0 diet had the highest REA, while those reared in the SPV20 diet had the lowest value.

### 3.4. In Vitro GP and CH_4_ Production

**Figure 1:**
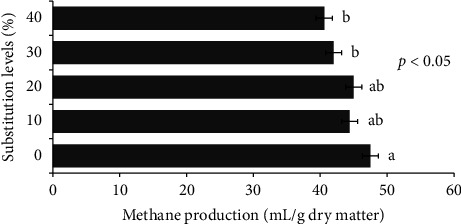
Response of methane production to increased substitution levels of concentrate mix with sweet potato vine.

**Figure 2:**
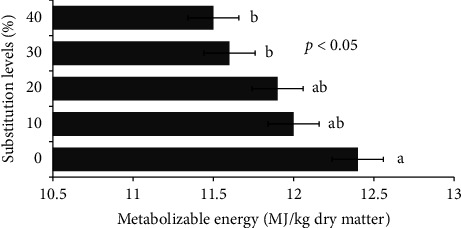
Effect of the substitution of concentrate mix with sweet potato vine on metabolizable energy.

The *in vitro* 24-h GP was higher (*p* < 0.001) for the SPV0 diet than for the SPV30 and SPV40 diets (Table 7). The GP was similar among the SPV20, SPV30, and SPV40 diets. The SPV showed the lowest (*p* < 0.001) 24-h GP as compared to treatment diets. The CH_4_ production of the SPV0 diet was similar to that of the SPV10, SPV20, and SPV30 diets. However, the SPV40 diet and SPV had the lowest CH_4_ production and differed (*p* < 0.001) from that of SPV0. The pCH_4_ did not differ among treatment diets but was higher (*p* < 0.01) than that observed in SPV. As shown in Figure 1, the *in vitro* CH_4_ production consistently and significantly reduced across the treatment diets as the substitution levels of CM with SPV increased. The ME in the SPV0 diet was similar to that in the SPV10 and SPV20 diets but was higher (*p* < 0.001) than that in the SPV30 and SPV40 diets and SPV. Both the SPV30 and SPV40 diets had higher (*p* < 0.001) ME values than the SPV. The dOM did not differ between treatment diets (Figure 2). Although SPV had the lowest dOM, it did not differ (*p* > 0.05) from the SPV30 and SPV40 diets (Table 7). The SPV had the lowest GP, CH_4_, pCH4, and ME values than all other treatment diets.

## 4. Discussion

### 4.1. Nutrient Composition of Experimental Diets and SPV

As the substitution level of CM with SPV increased, the concentrations of crude ash, CP, NDFom, ADFom, and ADL improved which is attributed to the high corresponding values of SPV. The CP value of SPV was comparable to the values reported in [10, 21] for the leaf part of the plant but was higher than that reported in [8] which was reported for local sweet potato leaves. According to [21], the CP values ranged from 20% to 28% for the leaves of four varieties of sweet potato with an average value of about 24%. In another study, the CP content of different SPV varieties (which included both the leaves and stem) was reported to be in the range of 15.5%–18.7% on DM basis [5].

The NDF and ADF values observed in the current study were higher than those reported in [3, 10]. The nutrient content of the SPV could be affected by the type of variety and soil in which it has been cultivated. Moreover, such variations in the concentrations of structured carbohydrates might be associated with the maturity stage of the vine and the type of variety used. High level of ADF is particularly associated with low digestibility of the feed which would apparently reduce the feed intake by animals.

The SPV used in this experiment showed higher values for the trace elements of Fe (1211 mg/kg DM) and Mn (114 mg/kg DM), and this observation is consistent with those reported in [8]. Both elements play key functions in rumen digestion, performance, fertility, and fitness [22, 23]. The current results may not necessarily show an oversupply of both microelements considering the fact that grazing ruminants in smallholder settings are not adequately supplemented with such minerals [24]. Thus, the current observation indicates that SPV could serve as a reliable source of trace mineral supplementation for grazing ruminants. With increased substitution levels of CM with SPV, the Ca concentration enhanced while that of P slightly reduced and this observation is in line with that of [8], which reported a relatively high Ca concentration with low *p* value in SPV.

### 4.2. Intake of Nutrients, Weight Gain, and Carcass Components

Although total DM intake was similar in all treatment groups, that of CP and ADF consistently and significantly enhanced with increased substitution levels of CM with SPV whereas the EE intake decreased. In agreement with the current observation, [25] reported that the intake of CP, NDF, and ADF improved with the increased supplementary levels of sweet potato silage.

The lack of significant difference in final body weight and body weight gain among rams fed with different substitution levels of CM with SPV may suggest that the treatment diets were comparable in the supply of nutrients during the growth of the yearling rams. However, the pattern of the weekly body weight (data not shown) showed a linear increase which suggests that replacing CM by SPV has enhanced the growth performance of yearling rams. This observation is similar to that reported in [25], which reported that improved growth performance was observed in lambs fed with various levels of SPV silage containing 70% vine and 30% tuber. In another study, the authors in [3] reported that SPV silage has improved diet digestibility and the retention of nitrogen in tropical cattle. Similarly, the authors in [25] reported a decreasing trend of FCR with increasing supplementation levels of SPV silage in local goats which might be associated with the quality of the feed which is composed of not only potato vines but also the tuber part that might be rich in carbohydrates.

Although the slaughter and hot carcass weight was higher in rams fed with the control diet (SPV0) than in those fed with different levels of SPV, it did not show a clear trend among treatment diets. For instance, the slaughter weight of rams fed with SPV0 did not differ from those of SPV40, and the hot carcass weight of SPV0 was not different (*p* > 0.05) from that of SPV30. Such observations are indeed very difficult to justify based on previous works in the literature. The weight of the lumbar, dressing percentage, and rib-eye muscle area were similar across treatment diets, which suggests that replacement of CM with SPV might be an option to feedlots where conventional feeds may not be affordable. Likewise, the weight of hindleg was similar among all rams fed with treatment diets except those in SPV40, which suggests the significance of SPV supplementation to poor-quality forages in smallholder settings.

The dressing percentage was not affected by the replacement of CM with the SPV. Consistent with the current findings, the authors in [12, 25] reported a significant improvement in dressing percentage in goats and lambs supplemented with SPV. Improvement in carcass traits is very important for the smallholders because the commercial value of carcass components from sheep is determined by a range of different features, notably the rib-eye area (area of the *Longissimus dorsi* muscle), which has been a research focus on a molecular level by using advanced technologies [19, 26, 27]. The rib-eye muscle area is significantly associated with the amount of muscle in the carcass lean meat yield [28, 29] indicating its significance in commercial marketing.

### 4.3. In Vitro GP and Methane Production

To the authors' knowledge, there is only limited information available reporting on the CH_4_ production potential of SPV or other parts of the plant, making it difficult to discuss the current findings with a view to published works in the literature. The CH_4_ production (mL/g DM) of the SPV30 and SPV40 diets was (*p* < 0.05) lower than that of the control diet (SPV0) which indicates that the replacement of CM with SPV has considerably reduced the CH_4_ production. The CH_4_ reduction ability of SPV was reported in [8] and described as a potential candidate in mitigating CH_4_ emissions from tropical livestock. The potential of the SPV in reducing CH_4_ as compared to CM might be associated with the higher amount of fiber fractions present in SPV. On the other hand, many researchers [30–32] have reported that high concentrations of bioactive plant secondary metabolites such as polyphenolic compounds may have the potential to hinder the CH_4_ production in the rumen though the possible mechanisms of their effects on rumen methanogenesis have not yet fully understood [33].

On top of that, the CH_4_ emissions from the ruminants can be mitigated through the proper selection of feed ingredients including unconventional feeds to be used in the formulation of diets by giving emphasis to their suitable combination ratios [34, 35]. In the current study, the CH_4_ production was consistently reduced with increased substitution levels of CM with SPV. This observation could be explained by the fact that within the concentrate feeding, soluble sugars produce more gas and CH_4_ than feeding with SPV that would contain low sugar as it has been evident in its relatively low energy density.

The present result suggests that the use of SPV as a supplement of tropical forages could play a pivotal role in mitigating CH_4_ production from the ruminants with positive effects on voluntary feed intake, growth performances, feed conversion efficiency, and some carcass components. This observation is supported by the findings of [3] who reported that feeding the SPV silage to the heifers has improved the diet digestibility as well as the retention of nitrogen by reducing the CH_4_ emissions per unit of digested feed.

With increased substitution levels of CM with SPV, the contents of NDF, ADF, and ADL increased while that of GP, ME, and dOM reduced. These observations are consistent with those of [36] who reported a negative correlation of NDF and ADF with ME and dOM in legume plants. Although no significant difference was noted in the growth of yearling rams, the ME concentration has significantly decreased with increased substitution of CM with SPV notably in the SPV30 and SPV40 diets which may suggest the necessity of provision of good energy source feeds. Moreover, using SPV as a sole feed resource or increasing its substitution levels in the CM diet may affect its palatability with possible determinantal effects on ME digestibility specifically with high rates of inclusion as observed in rams fed with the SPV40 diet. Although the SPV could be supplied to animals in the form of silage, the CP content could be reduced substantially [3]. Therefore, the dried form of SPV might be preferred for feeding ruminants to maintain its CP content and prevent the possible loss of other nutrients during the process of silage making. Furthermore, the dOM did not differ among treatment diets, suggesting that feeding the dried form of SPV could be beneficial in small ruminant nutrition.

## 5. Conclusion

The replacement of the CM with SPV did not affect the feed consumption, body weight gain, FCR, some carcass components, and the dOM. Moreover, the inclusion of SPV considerably improved the total CP intake. The current findings also underscore the significance of SPV in reducing CH_4_ production *in vitro*. Thus, the SPV can be used as an alternative protein supplement feed resource by substituting up to 40% of the CM for local sheep while being a potential forage in mitigating CH_4_ production. However, it would be worthwhile to note that the ME has reduced with increasing substitution levels of CM with SPV being notably lower in the SPV30 and SPV40 diets than those of the SPV0, SPV10, and SPV20 diets.

## Figures and Tables

**Table 1:** Proportion of feed ingredients used in the concentrate mix (CM) and their analyzed proximate compositions (g/kg DM).

Feed ingredients	Proportion in CM (%)	Ash	Crude protein	Ether extract	aNDFom	ADFom	ADL
Maize	40	18	87	72	98	39	19
Wheat bran	40	46	160	52	331	118	32
Line seed cake	19.5	84	280	130	231	159	60
Salt	0.5	—	—	—	—	—	—

*Note:* ADFom, acid detergent fiber on organic matter basis; aNDFom, neutral detergent fiber on organic matter basis after amylase treatment.

Abbreviation: ADL, acid detergent lignin.

**Table 2:** Analyzed proximate compositions (g/kg DM) of sweet potato vine (SPV) and experimental diets containing different levels of SPV.

Nutrients	SPV0	SPV10	SPV20	SPV30	SPV40	Grass hay	SPV
Ash	54.0	63.0	72.0	83.0	91.0	83.0	148
Crude protein	162	180	193	205	212	65.0	258
Ether extract	74.0	61.0	58.0	56.0	53.0	13.0	27.0
NDFom	244	265	269	269	278	639	326
ADFom	105	125	130	141	157	416	202
ADL	32.0	42.0	41.0	41.0	49.0	61.0	74.0

*Note:* ADFom, acid detergent fiber on organic matter basis; aNDFom, neutral detergent fiber on organic matter basis after amylase treatment; SPV0, control diet without SPV; SPV10, diet with 10% replacement of concentrate mix by SPV; SPV20, diet with 20% replacement of concentrate mix by SPV; SPV30, diet with 30% replacement of concentrate mix by SPV; SPV40, diet with 40% replacement of concentrate mix by SPV.

Abbreviations: ADL, acid detergent lignin; SPV, sweet potato vine.

**Table 3:** Analyzed major and trace mineral composition of grass hay, sweet potato vine (SPV), and experimental diets.

Minerals	SPV0	SPV10	SPV20	SPV30	SPV40	Grass hay	SPV
*Major minerals (g/kg DM)*							
Calcium	2.4	2.9	3.7	5.0	6.4	5.4	11.2
Phosphorous	6.8	6.5	6.5	5.9	5.5	1.3	4.4
Magnesium	3.3	3.1	3.2	3.2	3.2	2.6	3.4
Potassium	9.4	12.8	15.4	18.4	22.5	10.2	35.5
Sodium	2.5	2.3	1.8	1.7	1.2	3.9	0.4

*Trace minerals (mg/kg DM)*							
Copper	10.9	10.4	9.9	9.0	8.2	4.6	5.7
Iron	745	721	784	823	777	3048	1211
Manganese	102	103	106	106	105	582	114

*Note:* SPV0, control diet without SPV; SPV10, diet with 10% replacement of concentrate mix by SPV; SPV20, diet with 20% replacement of concentrate mix by SPV; SPV30, diet with 30% replacement of concentrate mix by SPV and SPV40, diet with 40% replacement of concentrate mix by SPV.

**Table 4:** Effect of substitution of concentrate mix with sweet potato vine on feed intake, live weight and feed conversion efficiency of yearling rams (kg/head).

Parameters	SPV0	SPV10	SPV20	SPV30	SPV40	SEM	*p* values			
							ANOVA	Linear	Quadratic	Cubic
Initial body weight	19.7	18.5	17.7	18.8	18.1	0.67	0.452	—	—	—
Final body weight	24.1	23.1	21.8	23.3	22.6	0.66	0.298	0.478	0.434	0.711
Total weight gain	4.45	4.59	4.10	4.47	4.53	0.34	0.445	0.638	0.517	0.960
Daily weight gain (g/head)	54.3	56.0	50.0	54.6	55.2	4.32	0.444	0.637	0.513	0.963
Total feed intake	75.0	75.0	73.4	74.5	76.2	1.21	0.632	0.548	0.231	0.502
Daily feed intake (g/head)	914	915	896	909	929	14.7	0.639	0.559	0.232	0.504
FCR (kg feed/kg gain)	17.2	16.9	18.4	18.0	17.1	1.42	0.546	0.545	0.590	0.806

*Note:* SPV0, control diet without SPV; SPV10, diet with 10% replacement of concentrate mix by SPV; SPV20, diet with 20% replacement of concentrate mix by SPV; SPV30, diet with 30% replacement of concentrate mix by SPV and SPV40, diet with 40% replacement of concentrate mix by SPV.

Abbreviations: FCR, feed conversion ratio; SEM, standard error of the mean.

**Table 5:** Intake of nutrients from hay, concentrate mix and sweet potato vine across treatment diets (g/head/day).

Nutrient intake	SPV0	SPV10	SPV20	SPV30	SPV40	SEM	*p* value
*Grass hay*							
Dry matter	507	512	502	506	527	4.35	0.741
Crude protein	34.7	34.8	33.8	34.1	35.4	0.28	0.728
Ether extract	6.83	6.89	6.76	6.81	7.09	0.06	0.739
NDF	335	339	332	335	348	2.78	0.737
ADF	218	220	216	218	227	1.91	0.722

*Concentrate mix*							
Dry matter	373^a^	331^b^	298^c^	257^d^	220^e^	26.9	< 0.001
Crude protein	62.4^a^	62.5^a^	59.5^ab^	55.3^b^	49.0^c^	2.54	< 0.001
Ether extract	28.5^a^	21.2^b^	17.9^bc^	15.1^c^	12.3^d^	2.80	< 0.001
NDF	94.0^a^	91.9^a^	82.9^b^	72.6^c^	64.3^d^	5.66	< 0.001
ADF	40.5^b^	43.4^a^	40.1^b^	38.0^bc^	36.3^c^	1.20	< 0.001

*Sweet potato vine*							
Dry matter	—	37.8^d^	75.5^c^	113^b^	151^a^	24.3	< 0.001
Crude protein	—	9.94^d^	19.9^c^	29.8^b^	39.8^a^	6.42	< 0.001
Ether extract	—	1.04^c^	2.08^bc^	3.12^ab^	4.16^a^	0.67	< 0.001
NDF	—	12.6^d^	25.1^c^	37.7^b^	50.3^a^	8.11	< 0.001
ADF	—	7.78^d^	15.6^c^	23.4^b^	31.1^a^	5.02	< 0.001

*Total intake*							
Dry matter	881	881	875	876	898	4.14	0.753
Crude protein	97.1^e^	107^d^	113^c^	119^b^	124^a^	4.70	< 0.001
Ether extract	35.3^a^	29.1^b^	26.7^bc^	25.0^bc^	23.5^c^	2.07	< 0.001
NDF	430	443	440	445	463	5.36	0.155
ADF	259^c^	272^bc^	272^bc^	279^b^	294^a^	5.70	0.004

*Note:* SPV0, control diet without SPV; SPV10, diet with 10% replacement of concentrate mix by SPV; SPV20, diet with 20% replacement of concentrate mix by SPV; SPV30, diet with 30% replacement of concentrate mix by SPV and SPV40, diet with 40% replacement of concentrate mix by SPV.

Abbreviations: ADF, acid detergent fiber; NDF, neutral detergent fiber; SEM, standard error of the man.

^a–e^Means between levels of substitution with different superscript letters are significant.

**Table 6:** Least square means of main carcass components of yearling rams fed different levels of sweet potato vein by replacing the concentrate mixture.

Carcass components (kg/head)	SPV0	SPV10	SPV20	SPV30	SPV40	SEM	*p* values			
							ANOVA	Linear	Quadratic	Cubic
Preslaughter weight	23.4^a^	19.9^b^	19.7^b^	19.5^b^	20.6^ab^	0.730	0.018	0.025	0.007	0.409
Hot carcass weight	8.78^a^	7.60^b^	7.71^b^	7.75^ab^	7.36^b^	0.242	0.007	0.003	0.154	0.013
Dressing percentage	37.6	38.5	39.1	39.7	35.8	0.931	0.108	0.489	0.022	0.205
Thorax	2.10^a^	1.72^b^	1.75^b^	1.81^b^	1.81^b^	0.06	0.010	0.031	0.007	0.047
Foreleg	1.59^a^	1.37^b^	1.37^b^	1.39^b^	1.31^b^	0.04	0.006	0.002	0.087	0.031
Hindleg	1.87^a^	1.65^ab^	1.73^ab^	1.72^ab^	1.55^b^	0.064	0.015	0.006	0.952	0.016
Lumbar	1.25	1.06	1.48	1.13	1.05	0.054	0.162	0.105	0.594	0.076
Neck	0.745	0.747	0.730	0.710	0.645	0.046	0.570	0.146	0.461	0.873
Liver	0.570^a^	0.487^ab^	0.450^ab^	0.537^ab^	0.417^b^	0.039	0.038	0.031	0.692	0.032
Heart	0.112	0.097	0.105	0.105	0.112	0.008	0.611	0.759	0.209	0.541
REA (cm^2^)	20.0	19.4	16.9	18.3	19.7	2.01	0.679	0.764	0.224	0.712

*Note:* SPV0, control diet without SPV; SPV10, diet with 10% replacement of concentrate mix by SPV; SPV20, diet with 20% replacement of concentrate mix by SPV; SPV30, diet with 30% replacement of concentrate mix by SPV and SPV40, diet with 40% replacement of concentrate mix by SPV.

Abbreviations: REA, rib-eye muscle area; SEM, standard error of the mean.

^a,b^Means between treatment diets with different superscript letters are significant.

**Table 7:** *In vitro* gas and methane productions, metabolizable energy and digestible organic matter contents of treatment diets and sweet potato vine (SPV).

Parameters	SPV0	SPV10	SPV20	SPV30	SPV40	SPV	SEM	*p* value
24-h GP (mL/g DM)	276^a^	262^ab^	260^ab^	241^b^	236^b^	174^c`^	5.310	< 0.001
CH_4_ (mL/g DM)	47.5^a^	44.4^ab^	45.0^ab^	42.1^ab^	40.6^b^	27.1^c^	0.934	< 0.001
pCH_4_ (%)	17.2^a^	16.9^a^	17.3^a^	17.4^a^	17.2^a^	15.4^b^	0.222	0.001
ME (MJ/kg DM)	12.1^a^	11.6^ab^	11.5^ab^	11.0^ab^	10.8^b^	8.69^c^	0.153	< 0.001
dOM (%)	74.8^a^	73.7^a^	74.3^a^	72.4^ab^	72.3^ab^	68.2^b^	0.941	0.007

*Note:* CH_4_, methane production; pCH_4_, percentage of methane production in GP; SPV0, control diet without SPV; SPV10, diet with 10% replacement of concentrate mix by SPV; SPV20, diet with 20% replacement of concentrate mix by SPV; SPV30, diet with 30% replacement of concentrate mix by SPV and SPV40, diet with 40% replacement of concentrate mix by SPV.

Abbreviations: DM, dry matter; dOM, digestible organic matter; GP, gas production; ME, metabolizable energy; SEM, standard error of the mean.

^a–c^Means between substitution levels with different superscript letters are significant.

## Data Availability

The data used to support the findings of this study can be available from the corresponding author upon rational request.
